# The application and progress of stem cells in auricular cartilage regeneration: a systematic review

**DOI:** 10.3389/fcell.2023.1204050

**Published:** 2023-07-26

**Authors:** Yu Liu, Wenqing Wu, Chun Seunggi, Zhengyong Li, Yeqian Huang, Kai Zhou, Baoyun Wang, Zhixing Chen, Zhenyu Zhang

**Affiliations:** ^1^ Department of Burn and Plastic Surgery, West China Hospital, Sichuan University, Chengdu, China; ^2^ Department of Plastic Reconstructive and Aesthetic Surgery, West China Tianfu Hospital, Sichuan University, Chengdu, China; ^3^ West China Hospital, Sichuan University, Chengdu, China

**Keywords:** stem cells, auricular cartilage, adipose-derived stem cells, bone marrow mesenchymal stem cells, perichondrial stem/progenitor cells, auricular reconstruction

## Abstract

**Background:** The treatment of microtia or acquired ear deformities by surgery is a significant challenge for plastic and ENT surgeons; one of the most difficult points is constructing the scaffold for auricular reconstruction. As a type of cell with multiple differentiation potentials, stem cells play an essential role in the construction of cartilage scaffolds, and therefore have received widespread attention in ear reconstructive research.

**Methods:** A literature search was conducted for peer-reviewed articles between 2005 and 2023 with the following keywords: stem cells; auricular cartilage; ear cartilage; conchal cartilage; auricular reconstruction, regeneration, and reparation of chondrocytes; tissue engineering in the following databases: PubMed, MEDLINE, Cochrane, and Ovid.

**Results:** Thirty-three research articles were finally selected and their main characteristics were summarized. Adipose-derived stem cells (ADSCs), bone marrow mesenchymal stem cells (BMMSCs), perichondrial stem/progenitor cells (PPCs), and cartilage stem/progenitor cells (CSPCs) were mainly used in chondrocyte regeneration. Injecting the stem cells into the cartilage niche directly, co-culturing the stem cells with the auricular cartilage cells, and inducing the cells in the chondrogenic medium *in vitro* were the main methods that have been demonstrated in the studies. The chondrogenic ability of these cells was observed *in vitro*, and they also maintained good elasticity and morphology after implantation *in vivo* for a period of time.

**Conclusion:** ADSC, BMMSC, PPC, and CSPC were the main stem cells that have been researched in craniofacial cartilage reconstruction, the regenerative cartilage performed highly similar to normal cartilage, and the test of AGA and type II collagen content also proved the cartilage property of the neo-cartilage. However, stem cell reconstruction of the auricle is still in the initial stage of animal experiments, transplantation with such scaffolds in large animals is still lacking, and there is still a long way to go.

## Introduction

Ear deformities can be classified as congenital microtia and acquired trauma, such as injury, burn, or skin cancer excision (Otto et al., 2015; Jessop et al., 2016). Microtia is usually associated with atresia or stenosis with conductive hearing loss (80% of cases) (Mussi et al., 2019). In children, microtia with hearing impairment may be associated with delayed language development, learning difficulty in school, and difficulty interacting with others (Billings et al., 2016; Zhu and Chen, 2016). Deformities and absence of an ear can also lead to negative psychological effects due to esthetic modification of the face, lack of symmetry, differences in the appearance of the ears, and functional issues, for example, wearing glasses. Not by accident, 55% of people with microtia reported low confidence, dissatisfaction, and depression, and 52% of the subjects showed signs of anxiety, which compromised their quality of life (Horlock et al., 2005; Li et al., 2010).

Ear reconstruction continues to be one of the biggest challenges for ENT and plastic surgeons, regardless of whether it involves total auricular reconstruction for congenital microtia or auricular traumatic defect reparation (Wilkes et al., 2014). The auricle is one of the most complex three-dimensional structures in the human body, so being able to construct a satisfactory, complete outer ear has been a difficult goal for many years. In developed countries, there are more than a million patients who undergo some kind of operation involving cartilage reconstruction every year (Chang et al., 2003). However, adult human cartilage shows poor capability for repair and regeneration; at the same time, lack of blood vessels on the surface and inside of the cartilage also limits the survival of the cartilage itself and the skin on the cartilage surface (Dyson et al., 2019).

In recent years, several surgical procedures have been developed for repairing cartilage defects, which highly depend on technique and are limited to small areas of lesions (Ciorba and Martini, 2006). As for huge defects and microtia, simple surgical repair has been unable to meet their therapeutic requirements. The three mainstream treatment strategies are shown as follows: 1) silicone ear prostheses fixed through osseointegrated implants or adhesive; 2) auricular reconstruction with the synthetic material implant; 3) auricular reconstruction with autologous costal cartilage (Narges Baluch et al., 2014).

To enhance the strength of the implanted synthetic materials and the regeneration ability of cartilage, several kinds of stem cells were used in these scaffolds. Since cartilage has a very slow turnover at cellular and molecular levels, it has limited capability for self-renewal and self-repair. Cartilage tissue is complex and consists of chondrocytes and a cartilage-specific extracellular matrix (which is mainly composed of collagens and proteoglycans) (Ciorba and Martini, 2006). Adult stem cells/progenitor cells were first identified by Till and McCulloch (1961). These cells can produce multiline hematopoietic colonies in the spleen. The concept of mesenchymal stem cells (MSCs) equal to adult stem cells was first proposed by Caplan in 1991 (Ai, 1991) based on the early research results of Friedenstein and Gerasimov (1987). However, stem cells did not attract global attention until Pittenger et al. (1999)’s multilineage study found non-hematopoietic stem cells capable of multilineage differentiation. Since the original identification of MSC differentiation, its potential has expanded, (Spencer et al., 2021) and since the initial identification of bone marrow-derived MSC/progenitor cells, MSC/progenitor cells have also been identified in tendon (Bi et al., 2007), articular cartilage (Williams et al., 2010), auricular cartilage (Xue et al., 2016), trachea cartilage (Moshkbouymatin, 2019), ligament (Lee et al., 2019), fat (Rodeheffer et al., 2008), and muscle tissue (Mitchell et al., 2010).

Once the progenitor cells/stem cells have been isolated and expanded, the stem cells need to begin to differentiate into the target tissue. Differentiation into chondrocyte-“like” lineages has been achieved for more than 30 years, and there is a wide range of prospective not only put optimal growth factors but also on the use of mechanical conduction (Humphries et al., 2022). This review is to demonstrate the mechanisms of different kinds of stem cells in auricular reconstruction and auricular cartilage reparation.

## Limitations of current techniques

In regular auricular reconstruction surgery, the prevailing gold standard requires three or four autologous costal cartilage segments that were harvested ipsilaterally or contralaterally (Thomson et al., 1995). Because of the poor regeneration ability of cartilage, the integrity and stability of the chest are damaged by such procedures. In these conditions, abnormally shaped ribs move backward under the force of respiratory muscles and negative thoracic pressure, leading to a local depression on the chest, especially in patients in the growth and development period (Park, 1997). Donor site morbidities were reported to be pneumothorax, atelectasis, pleural effusion, etc., at an early stage (Thomson et al., 1995; Kim et al., 2011; Moon et al., 2012), and in the delayed stage, the morbidities were described as persistent pain, thoracic scoliosis, clicking, seroma, abnormal scarring, contour deformity, etc. (Osorno, 1999; Osorno, 2007; Uppal et al., 2008; Moon et al., 2012). Although perioperative procedures are optimized to help reduce donor site morbidities, these problems remain incompletely solved (Walton and Beahm, 2002).

The reconstructed costal cartilage scaffold is different from normal auricular cartilage in terms of its mechanical properties (Brent, 1999). The skin tension of the flap leads to additional morphological distortion, and skin flap necrosis or postoperative infections also cause extrusion of the cartilage scaffold (Brent et al., 1992; Firmin, 1998). In addition, the calcification and resorption of costal cartilage after transplantation result in the stiffness and thickness of the scaffold, along with an indistinct contour and discomposed shape (Berghaus and Toplak, 1986; Firmin, 1998; Brent, 1999; Mori et al., 2002; Walton and Beahm, 2002)

Synthetic materials such as high-density porous polyethylene or Medpor (Porex Surgical, Inc., College Park, GA) were used in clinical treatment to eliminate donor site morbidities and achieve a durable shape. However, the immune response induced by the Medpor scaffold leads to a significantly higher rate of exposure than that of the autologous costal cartilage scaffold (Zhao et al., 2009). In terms of the esthetic outcomes of porous polyethylene and costal cartilage constructs, the former was superior in definition, shape, and size match but inferior in protrusion, location, and color (Constantine et al., 2014).

To decrease the risk of immune rejection, autologous cells are suggested in clinical application of tissue engineering. In auricular reconstruction, regenerating a full-size human auricular scaffold requires over 200 million isolated cells (Reiffel et al., 2013), which is impossible for microtia patients with minimal ear cartilage remnants. *In vitro* expansion of chondrocytes often results in dedifferentiation, showing an enhanced behavior of fibroblasts (deposition of type I collagen and reduction of cartilage matrix deposition such as type II collagen), which leads to a significant reduction in cartilage elasticity and mechanical strength (Schnabel et al., 2002; Mandl et al., 2004; Bichara et al., 2012; Reiffel et al., 2013; Cohen et al., 2016). Additionally, a 3D construct culture is required prior to implantation of monolayer containing expanded chondrocytes (Zhou et al., 2018).

Therefore, inducing the MSCs or progenitor cells (PCs) into chondrocytes is expected to alleviate the burden of microtia chondrocyte requirement (Yamamoto et al., 2004; McCorry et al., 2016; McCorry and Bonassar, 2017). MSC-derived auricular scaffolds should be paid attention to as a development trend for ear reconstruction in the future.

## Methods

**FIGURE 1:**
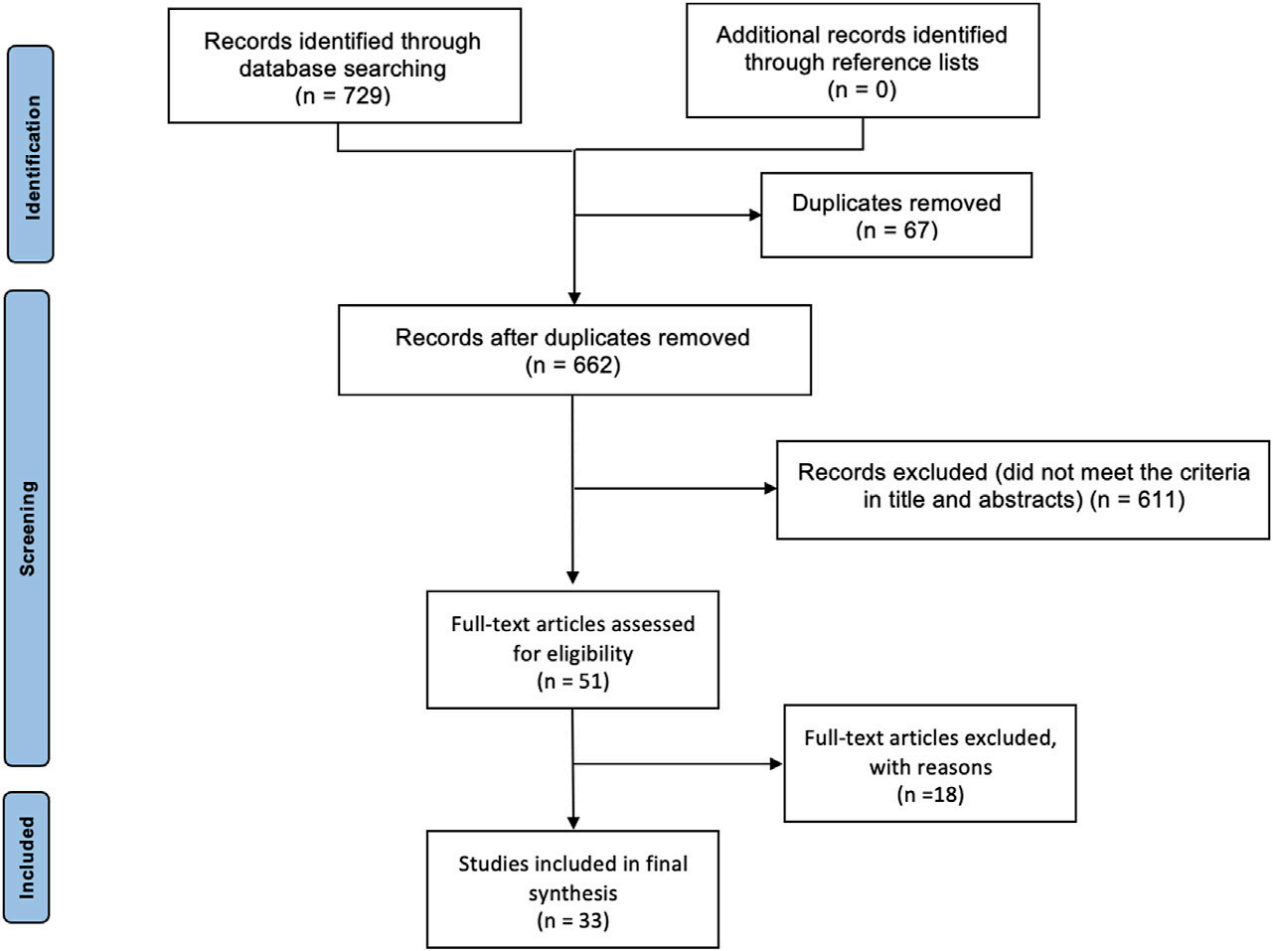
PRISMA flow diagram.

PubMed, MEDLINE, Cochrane, and Ovid databases were searched from 2005 to 2023 using the following key terms: stem cells; auricular cartilage; ear cartilage; conchal cartilage; auricular reconstruction, regeneration, and reparation of chondrocytes; tissue engineering. Across these databases, these search terms produced 729 results. After removing the duplicates, 662 results were reserved, and then we excluded those articles that were not in English and where the main interest in cartilage was not auricular. Inclusion criteria were studies focused on stem cell application in auricular reconstruction. Titles were screened and removed if not relevant. Abstracts were then screened and taken forward for full-text review, if appropriate. Finally, 33 articles focused on the topic were included in this review (Figure 1).

## Stem cell types in chondrogenesis

Thirty-three research articles were selected, and the main characteristics were summarized. Four sorts of mainstream stem cells, i.e., adipose-derived stem cells, bone marrow mesenchymal stem cells, perichondrial stem/progenitor cells, and cartilage stem/progenitor cells, were generally applied to reconstruct the auricular cartilage. Furthermore, different cultures and inducement methods can present different results of chondrogenesis.

### Adipose-derived stem cells

Adipose tissue is derived from the mesoderm during embryonic development and is present in all mammals throughout the body. Although two types of adipose tissue (brown and white) exist, white adipose tissue gives rise to the commonly studied ADSCs. The methods for isolating ADSCs include enzymatic digestion, mechanical separation, and tissue adherence. Enzymatic digestion is the most commonly used method, which uses various enzymes such as collagenase, trypsin, and hyaluronidase to digest the adipose tissue and isolate ADSCs (Minteer et al., 2013). ADSCs have multipotent differentiation potential and can differentiate into osteoblasts, chondrocytes, myocytes, and adipocytes. In addition, they have self-renewal and proliferation abilities and have a wide range of applications. Commonly used markers for ADSCs include CD29, CD44, CD73, CD90, and other mesenchymal stem cell markers, as well as CD34, CD45, and other markers for vascular endothelial and hematopoietic stem cells. These markers can help identify and purify ADSCs (Patricia et al., 2002).

According to the former study, it demonstrated that ADSCs are relatively easy to obtain (from adipose tissue) and to culture, and their extraction is less invasive in comparison with BMMSCs. (Raghunath et al., 2005). Based on these characteristics, ADSCs may represent an excellent source for cell therapy (Patricia et al., 2002; Yamamoto et al., 2007). The main characteristics of the studies focused on ADSC differentiation are analyzed and reported in Table 1.

**TABLE 1:** Main characteristics of the eight studies included in the review on adipose-derived stem cells.

Studies	Species of cells		Sorts of cells	Recipient animal	*In vitro*/*in vivo*	Scaffold	Definition of auricular cartilage differentiation	Conclusion
Bahrani et al. (2012)	Rabbit		ADSCs	Rabbit	*In vivo*	—	Histopathological examination (HE^c^)	Under specialized *in vivo* conditions favoring repair with a specific cell maturation line, ADSCs can proliferate and differentiate into chondrocytes, enabling chondrogenesis and cartilage defect repair
Chen et al. (2022)	Human		ADSCs	Nude mouse	*In vivo*	GelMA hydrogel	Histopathological examination (HE)	A single dose of ADSC-engineered exosomes was efficacious for tissue-engineered cartilage regeneration
Landau et al. (2021)	Human		ADSCs; ACCs^a^	Nude mouse	*In vitro*/*in vivo*	PCL	Histopathological examination (Safranin-O, alcian-blue, and HE)	PCL scaffolds loaded with patient-derived chondrocytes produced from either auricular cartilage or costal cartilage biopsies combined with adipose-derived mesenchymal stem cells. Cartilage formation was measured within the construct *in vitro*, and cartilage maturation and stabilization were observed 12 weeks after its subcutaneous implantation into a murine model
Leslie et al. (2018)	Rabbit		ADSCs	Rabbit	*In vivo*	—	EPIC-microCT and histopathological examination (HE)	Multipotent ADSCs formatted in alginate microbeads 200 μm diameter can be delivered to auricular cartilage defects to stimulate chondrogenesis
Lin et al. (2017)	Human		ADSCs	Nude mouse	*In vivo*	Small intestine submucosa	Histopathological examination (HE; Alizarin Red S) and immunohistology (CD31, h-MHC)	The authors’ study found that the combination of human adipose stem cells and small intestine submucosa could provide a more durable ear-shaped construct *in vivo*
Oh et al. (2020)	Rabbit		ADSCs	Rabbit	*In vivo*	—	Histopathological examination (HE and Masson) and immunohistology (S-100)	ADSCs have beneficial effects, but the secretome has no significant impact on the auricular cartilage regeneration. Therefore, ADSCs might be more effective treatment than their secretome in the repair of auricular cartilage defects
Oh et al. (2018)	Rabbit		ADSCs	Rabbit	*In vivo*	—	Histopathological examination (HE and Masson) and immunohistology (S-100)	ASC treatment has a regenerative effect on auricular cartilage defects in rabbits, which is characterized by new cartilage formation composed of chondrocytes and cartilage-specific ECM at the site of the surgically created defect with stronger expression of S-100 protein and higher expression of type II collagen and TGF-β1
Xu et al. (2018)	Controlled	ADSCs, PRF^b^		Rabbit	*In vivo*	—	Histopathological examination (HE) and fluorescent Dil dye	Allogenic ADSCs in combination with PRF can accelerate regeneration in full-thickness cartilage defects in the rabbit ear model without causing a significant immune response

^a^ ACCs: auricular cartilage cells.

^b^ PRF: platelet-rich fibrin.

^c^ HE: hematoxylin and eosin.

As we already know, ADSCs have multi-directional differentiation potential, and in different environments, they can be induced into different kinds of cells. ADSCs have been identified as novel potential candidates for the reconstruction of cartilage defects *in vivo*. In Bahrani’s study, rabbit ADSCs were isolated and cultured to passage 3; these stem cells were harvested and injected into the area where the auricular cartilage was removed by surgery, and then they found that the defects were repaired by neo-cartilage, which was differentiated from ADSCs (Bahrani et al., 2012). In another study, Leslie et al. (2018) found that rabbit ADSCs in alginate microbeads could regenerate auricular cartilage when they were placed in the auricular defect areas, but were not fully integrated with the surrounding tissues. In chondrogenic media, ADSCs expressed mRNAs for aggrecan (AGA), type II collagen, and type X collagen, which means these newborn cells have the characteristics of ear cartilage. Applying the same condition, Landau et al. (2021) used the 3D-printed clinical-grade polycaprolactone scaffold loaded with patient-derived chondrocytes generated from auricular cartilage or costal cartilage biopsy combined with ADSCs. Chondrogenesis potential was measured *in vitro*, and cartilage maturation and stability were observed 12 weeks after subcutaneous implantation in the mouse model. In the study by Se-Joon Oh et al. (2020), published in 2018 (57), rabbit ADSCs were injected into rabbit auricular defects; after 1 month, histopathology showed islands of new cartilage formation at the site defects. At the same time, the expressions of collagen type II and TGF-β1 were significantly higher in the ADSCs than in the blank control group. Except for this, in the other research studies, they contrasted the therapeutic efficacies of ADSCs and their secretome in terms of rabbit auricular cartilage regeneration, and they found that ADSCs could significantly enhance new cartilage formation, but their secretome did not. Therefore, ADSCs may be more effective in the repair of auricular cartilage defects. In the aforementioned studies, the main mechanism of ADSC differentiation into ear cartilage is that ADSCs can differentiate into ear cartilage in a large amount in a cartilage environment or chondrogenic medium.

However, in Chen et al.’s (2022) study, in contrast to Se-Joon Oh’s study, they isolated and purified the exosomes from human ADSCs, co-cultured them with microtia chondrocytes in Gelma hydrogel, and then implanted the tissue-engineered cartilage into subcutaneous pockets of nude mice for 6 weeks. Finally, they found that a single dose of Engineered exo was efficacious for tissue-engineered cartilage regeneration. Engineered exo effectively promoted the proliferation, survival, and mature cartilage formation of microtia chondrocytes through the hsa-miR-23a-3p/PTEN/PI3K/AKT/mTOR axis. They also designed Engineered exo by directly transfecting agomir-23a-3p into parental passage 4 ADSCs to isolate exosomes enriched with hsa-mir-23a-3p and optimize favorable effects on the cell viability and new cartilage formation.

In addition to direct injection of ADSCs into the cartilage site and culturing of ADSCs in chondrogenic medium, Lin et al. (2017) cultured and induced human ADSCs in the small intestine submucosa scaffold, which differentiated into osteocytes, but not chondrocytes. The ear-shaped human ADSCs/small intestine submucosa construct could maintain the shape *in vivo* for up to 1 year; angiogenesis was evident in human ADSCs/small intestinal submucosal structures at 6 months and persisted for 1 year; and the mechanical properties were highly equal to those of the natural auricular cartilage. Xu et al. (2018) evaluated the efficacy of allogeneic ADSCs combined with platelet-rich fibrin (PRF) in the treatment of ear cartilage defects in rabbits and found that ADSCs/PRF could promote the regeneration of full-thickness cartilage defects in rabbit ears without causing an obvious immune response. The results showed that allogeneic ADSCs plus PRF could be successfully used for cartilage regeneration.

### Bone marrow mesenchymal stem cells

BMMSCs are a type of multipotent stem cells that were first discovered in the 1970s. BMMSCs can differentiate into various cell types, including osteoblasts, chondrocytes, adipocytes, and myocytes (Prockop, 1997). BMMSCs also produce many biologically active molecules, such as cytokines, growth factors, and extracellular matrix, which have important biological functions, such as stimulating cell proliferation, repairing tissue damage, and anti-inflammatory effects. The isolation methods of BMMSCs mainly include the adherent method and the density gradient centrifugation method. The adherent method refers to culturing bone marrow cells in plastic culture bottles, allowing MSCs to adhere to the bottle wall, and then removing non-adherent cells to leave MSCs. The density gradient centrifugation method separates MSCs from other cells by centrifugation in a density gradient centrifuge tube (Bianco et al., 2008). The markers of BMMSCs include CD73, CD90, and CD105. These markers are surface markers of MSCs and the main identification criteria for MSCs. In addition, BMMSCs also express some embryonic stem cell markers, such as Oct-4 and Nanog, indicating that BMMSCs have stem cell characteristics (Dominici et al., 2006; Jia et al., 2018).

In our review, BMMSCs might show better effects than ADSCs in auricular cartilage regeneration, and more research was conducted in the direction of BMMSCs. BMMSCs are one of the most important cells for repairing cartilage defects *in vivo* and have the characteristics of multipotent differentiation. *In vitro*, BMMSCs can be purified and cultured, have a durable phenotype and cellularity, and induce chondroblasts to secrete the cartilage matrix (Erickson et al., 2002; Jiang et al., 2002; Solchaga et al., 2004; Wagers and Weissman, 2004). The main characteristics of the studies focused on BMMSC differentiation are analyzed and reported in Table 2.

**TABLE 2:** Main characteristics of the 14 studies included in the review on bone marrow mesenchymal stem cells.

Studies		Species of cells			Sorts of cells			Recipient animal			*In vitro*/*in vitro*			Scaffold		Definition of auricular cartilage differentiation			Conclusion			
Hassan et al. (2022)		Rabbit			ADSCs, BMSCs, ESCs^a^			Rabbit			*In vivo*			—		Histopathological examination (HE^i^, Masson, toluidine blue, and orcein) and immunohistology (S-100)			BMMSCs had the highest proliferation rate and chondrogenic potential compared to ADSCs and ESCs, as shown in histological assessments, with better reactivity of the S-100 protein and higher production of col II, aggrecan, and TGF-b1, which could be of superior value over ADSCs and ESCs for the regeneration of the cartilaginous defects			
Cheng et al. (2014)		Rabbit			BMMSCs			Rabbit			*In vivo*			PLGA^b^		Histopathological examination (HE)			BMMSCs can be used as seed cells to repair cartilaginous defects in the head and neck through cartilage tissue engineering and shed light on the potential of the application of BMMSCs in clinical cartilage tissue engineering			
Cohen et al. (2018)		Human			BMMSCs, ACCs^c^			Nude mouse/Rat			*In vivo*			Collagen hydrogel		Histopathological examination (Safranin O/Fast green, picrosirius red, and Verhoeff’s/Van Gieson)			The successful engineering of a patient-specific human auricle using exclusively human cell sources without extensive *in vitro* tissue culture prior to implantation, a critical step toward the clinical application of tissue engineering for auricular reconstruction			
Dong et al. (2022)		Human			BMMSCs, ACCs			Nude mouse			*In vivo*			PLA^d^		Histopathological examination (HE, Safranin O/fast green, and Verhoeff’s/Van Gieson)			Co‐implantation of ACCs and BMMSCs a ratio as low as 1:9 of ACCs to BMMSCs within a Type I collagen matrix generates clinically relevant sized cartilage indistinguishable from that of native auricular cartilage upon gross, histologic, and biomechanical analysis after 6 months *in vivo*			
Hou et al. (2022)		Rabbit			BMMSCs			Pig			*In vivo*			—		Histopathological examination (HE and Safranin O/fast green) and immunohistology (PRG4 and α-SMA)			The current study demonstrated that the *in situ* native cartilage niche is the determining factor for the ultimate regenerated cartilage type of stem cells and chondrocytes. It can regulate the directional differentiation of stem cells and transdifferentiation of chondrocytes to regenerate a specific type of cartilage consistent with the native niche			
Kang et al. (2012)		Pig			BMMSCs, ACCs			Nude mouse			*In vivo*			PGA^e^/PLA		Histopathological examination (HE and Safranin O) and immunohistology (collagen type II, delta-like1/fetal antigen1, and Ki67)			The hypertrophy and mineralization of engineered cartilage in the approach of BMSC chondrogenic induction were found to be consistent with the upregulation of RUNX2 and downregulation of SOX9. Moreover, the approach of co-culturing BMMSCs and auricular chondrocytes reduced the hypertrophy, enhanced the elastic modulus, and improved the chondrogenic and proliferative potentials of engineered cartilage			
Kang et al. (2013)		Pig			BMMSCs, ACs^f^			—			*In vitro*			PGA		Histopathological examination (HE and Safranin O) and immunohistology (collagen type II)			As few as 30% of chondrocytes could be used as seeding cells for the construction of cartilage with a satisfactory shape and quality when co-cultured with BMMSCs			
Karimi et al. (2016)		Human			BMMSCs			Nude rat			*In vivo*			—		Histopathological examination (HE)			Using the ear cadaver framework seeded with bone marrow stem cells for reconstruction of ear is a feasible, fast, 1-stage technique and the elasticity, shape, size, and weight of the framework would be preserved			
Morrison et al. (2016)		Calf			BMMSCs, ACCs			Nude mouse			*In vivo*			Collagen hydrogel		Histopathological examination (Safranin O/fast green and Verhoeff’s/Van Gieson)			We demonstrate a clinically translatable cell-sourcing strategy to fabricate elastic cartilage using only half the number of auricular chondrocytes normally required			
Pleumeekers et al. (2015)		Calf, human			BMMSCs, ACCs			Nude mouse			*In vivo*			—		Immunohistology (collagen type II)			This study demonstrates that constructs containing a combination of 80 percent human bone marrow-derived mesenchymal stem cells and 20 percent bovine ear or nasal chondrocytes produced similar quantities of cartilage matrix components as constructs containing only chondrocytes			
Posniak et al. (2022)		Human			BMMSCs, ACCs			—			*In vitro*			—		Histopathological examination (HE and toluidine blue)			These results showed that the combination of MSCs and ACCs can yield cell proliferation similar to that of MSC controls. Simultaneously, the combination of MSCs and ACCs produces chondrogenic expressions that match that of ACCs controls			
Zhang et al. (2014)		Human, goat			BMMSCs, ACCs			Nude mouse			*In vivo*			PGA		Histopathological examination (HE and Safranin O) and immunohistology (collagen type II)			Regenerative technology of human-ear shaped cartilaginous tissue based on MCs and stem cells with lower cost and more stable cartilage formation was successfully established by the co-transplanting strategy, which provided a promising strategy for clinical translation of the engineered human-ear shaped cartilaginous tissue			
Zhao et al. (2017)		Sheep			BMMSCs, ACCs			Nude mouse			*In vivo*			Collagen		Histopathological examination (HE, toluidine blue, and Safranin O) and immunohistology (collagen types I and II)			Chondrocyte-conditioned medium had a stronger influence on chondrogenesis than supplementation of the standard culture medium with TGF-β3 without inducing calcification			
Otto et al. (2018)		Horse			BMMSCs, ACCs, PPCs^g^			—			*In vitro*			GelMA hydrogel		Histopathological examination (HE and Safranin O/fast green) and immunohistology (collagen types I, II, and VI)			Although under the current culturing conditions, bone marrow derived MSCs seemed to perform better in terms of matrix production, major advantages of ACPCs^h^ include the ability to generate high cell numbers, upregulation of the elastin gene, and a limited endochondral ossification potential			

^a^ ESCs, ear stem cells.

^b^ PLGA, poly (dl-lactide-co-glycolide).

^c^ ACCs, auricular cartilage cells.

^d^ PLA, polylactic acid.

^e^ PGA, polyglycolic acid.

^f^ ACs, articular cartilage cells.

^g^ PPCs, perichondrial progenitor cells.

^h^ ACPCs, auricular cartilage progenitor cells.

^i^ HE, hematoxylin and eosin.

Although BMMSCs can differentiate into a variety of cells, they need certain circumstances that can cause their induction in target cells. In many studies, auricular cartilage cells were co-cultured with BMMSCs to obtain a significant number of cartilage cells for auricular reconstruction. In Zhang et al. (2014)’s study, they harvested and co-cultured the human microtia cartilage cells (MCs) and goat BMMSCs and implanted the cells and ear-shaped scaffolds in nude mice. After 12 weeks, a human-ear-shaped cartilaginous tissue with delicate structure and proper elasticity was successfully constructed. It shows that BMMSCs co-cultured with MCs would require fewer cartilage cells and construct a more stable scaffold than before. Moreover, the same results were also demonstrated in Karimi et al. (2016) and Morrison et al. (2016); they concluded that such an innovative cell sourcing strategy facilitates the efforts to achieve clinical translation of high fidelity. Kang et al. (2012); Kang et al. (2013) also co-cultured BMMSCs and chondrocytes in different ratios, and finally, the study found that 30% of chondrocytes are required to generate cartilage tissue of satisfactory shape and quality at least, and the MC: BMMSC ratio of 5:5 showed the highest Young’s modulus and the densest elastic fibers, which were consistent with the expression of DCN and LOXL2 genes (cartilage matrix-related genes). Nevertheless, in the study by Dong et al. (2022), they co-cultured human MCs and human BMMSCs and efficiently produced well-shaped human elastic cartilage without volume loss, even when human MCs accounted for only 10% of the total number of transplanted cells. In Cohen et al. (2018)’s study, they also found that BMMSCs and MCs co-cultured in a 1:1 ratio appeared as bundles of collagen fibers in the perichondrial layer, rich in proteoglycan deposits, forming an elastin fiber network similar to natural human ear cartilage, with the protein composition and mechanical stiffness of natural tissue. In Posniak et al. (2022)’s and Dong et al.’s study, they concluded that BMMSCs applied to replace auricular cartilage alleviate the requirement for large cartilage biopsies, which would otherwise be needed for sufficient cell numbers. To analyze the contribution of different cells in the co-culture system, Pleumeekers et al. (2015) used a xenogeneic co-culture system that included human BMMSCs and bovine ear chondrocytes or nasal chondrocytes in an 80:20 ratio. Based on these conditions, BMMSCs were found to play a trophic role in the co-culture system because aggrecan was expressed only by the chondrocytes.

Another strategy to obtain large-volume auricular chondrocytes is to induce BMMSCs in a chondrocyte induction medium (CM). BMMSCs were isolated from living bodies and amplified in CM *in vitro*. Cheng et al. (2014) confirmed these cells as chondrocytes and then implanted the cells onto a poly (D-L-lactide-co-glycolide) (PLGA) scaffold. After being cultured *in vivo* for 18 weeks, gross observation indicated that the cartilaginous defects were completely repaired by chondrocytes with smooth surfaces and similar color to the surrounding tissue. In Otto et al. (2018)’ s study, they cultured the BMMSCs in gelatin methacryloyl (gelMA). BMMSCs outperformed other cartilage-derived cell types in terms of matrix production and mechanical properties. Zhao et al. (2017) compared the different influences of BMMSCs in standard culture medium (SM) and CM for inducing BMMSCs. After amplifying for three passages *in vitro*, the cells were transplanted onto fibrous collagen scaffolds and precultured for 2 weeks, with or without transforming growth factor-beta 3 (TGF-β3). As shown in the results, after 12 weeks of *in vivo* culture, COL2A1 expression was upregulated in the CM compared to SM, and abundant neocartilage formation was observed in the implants that had been cultured in the CM, with or without TGF-β3. However, little cartilage matrix formation was observed in the SM group, regardless of the presence or absence of TGF-β3. It means that the effect of the CM on chondrogenesis was even stronger than that of the SM supplemented with TGF-β3, and there was no sign of endochondral osteogenesis.

For auricular cartilage reparation by injecting BMMSCs into the cartilage defect areas, Hassan et al. (2022) found that the auricle defects of the BMMSC group appeared completely healed with smooth surfaces and similar tissue color, and the treatment effects of BMMSCs were even better than those of ADSCs in their study. To research whether the anatomical location of cartilage could influence the differentiation of BMMSCs, Hou et al. (2022) transplanted the BMMSCs into native auricular and articular cartilage niches and found that the native cartilage niches were able to regulate BMMSC regeneration of elastic and hyaline cartilage despite the type of transplanted cartilage in these niches.

### Perichondrial stem/progenitor cells

In spite of ADSCs and BMMSCs, another kind of stem cells was also experimented by researchers, which could be isolated from perichondrial named PPCs. Unlike CSPCs, the perichondrium is vascularized and innervated.

PPCs within the perichondrium have been described as proliferating more rapidly than mature chondrocytes and being able to differentiate into other mesenchymal tissues under specific conditions (Togo et al., 2006). PPCs are multipotent stem cells derived from the perichondrium, with good proliferation and differentiation potential, capable of differentiating into chondrocytes, osteoblasts, myocytes, adipocytes, and other cell types (Ho et al., 2022). The isolation of perichondrial stem/progenitor cells is mainly achieved by mechanical separation and enzymatic digestion methods, such as enzymatic digestion and collagenase digestion. PPCs can differentiate into multiple cell types and have good proliferation and differentiation potential. They can be cultured for a long time *in vitro* and *in vivo*. The markers of the cells include CD105, CD90, CD73, and other mesenchymal stem cell markers, as well as CD146, CD271, Stro-1, and other stem cell markers. In addition, they also express some chondrocyte-related markers such as Sox9 and Col2a1 (Arai et al., 2002).

Compared with BMMSCs, auricular cartilage PPCs are easily separated from a donor site without ectopic tissue formation, such as calcifications or fibrous tissue formation. The main characteristics of the studies concentrating on the PCC differentiation analyzed are reported in Table 3.

**TABLE 3:** Main characteristics of the seven studies included in the review on perichondrial stem/progenitor cells.

Studies	Species of cells	Sorts of cells	Recipient animal	*In vitro*/*in vitro*	Scaffold	Definition of auricular cartilage differentiation	Conclusion
Derks et al. (2013)	Pig	ePPCs^a^, tPPCs^b^	Pig	*In vitro*	—	Histopathological examination (HE^d^, pentachrome, and alcian blue stain) and immunohistology (collagen type II)	Due to a high proliferative activity and a high chondrogenic capacity, ePPC might be a suitable cell source for cartilage tissue engineering
Kagimoto et al. (2016)	Monkey	PPCs^c^	Monkey/nude mouse	*In vivo*	—	Histopathological examination (Blyscan assay, HE, alcian blue, and Elastica van Gieson stain) and immunohistology (collagen type II)	The autologous transplantation of cartilage progenitors is potentially effective for reconstructing elastic cartilage
Oba et al. (2022)	Human	PPCs, ACCs	Nude mouse	*In vivo*	—	Histopathological examination (Blyscan assay, HE, alcian blue, and Elastica van Gieson stain) and immunohistology (collagen types I and II)	We succeeded in developing human auricular perichondrial chondroprogenitor cell-derived elastic cartilage *in vitro* that exhibits superficial effects when transplanted craniofacially, without major post-transplantation shrinkage
Togo et al. (2006)	Rabbit	PPCs, BMMSCs	Nude mouse	*In vitro*/vivo	Collagen sponge	Histopathological examination (toluidine blue and Elastica van Gieson) and and immunohistology (collagen type II)	Rabbit bone marrow mesenchymal stem cells used as controls could regenerate significantly smaller cartilage than perichondrocytes in the implant study
Xue et al. (2016)	Pig	PCCs, CSPCs	—	*In vitro*	—	Histopathological examination (toluidine blue)	We isolated cell populations from auricular cartilage and perichondrium and confirmed their stem cell properties by expression of stem cell surface marker, colony forming assay, and multiple differentiation potential
Zhang et al. (2019)	Pig	PCCs, CSPCs	—	*In vitro*	—	Histopathological examination (toluidine blue)	CSPCs showed a significant advantage in chondrogenesis *in vivo* with upregulated chondrogenic genes, a stable cartilage phenotype, and good mechanical properties
Otto et al. (2018)	Horse	BMMSCs, ACCs, PPCs	—	*In vitro*	GelMA hydrogel	Histopathological examination (HE and Safranin O/fast green) and immunohistology (collagen types I, II, and VI)	Although under the current culturing conditions, bone marrow-derived MSCs seemed to perform better in terms of matrix production, major advantages of ACPCs include the ability to generate high cell numbers, upregulation of the elastin gene, and a limited endochondral ossification potential

^a^ ePPCs: ear perichondrial progenitor cells.

^b^ tPPCs: tracheal perichondrial progenitor cells.

^c^ PPCs: perichondrial progenitor cells.

^d^ HE: hematoxylin and eosin.

The presence of progenitor cells in the auricular perichondrial was first proved in Togo et al. (2006)’s study; they compared the adipogenic and osteogenic ability of rabbit PPCs, cartilage stem cells (CSCs), and BMMSCs, and progenitor cells and stem cells were implanted into the dorsum of nude mice with a collagen sponge scaffold. The results demonstrated that the adipogenic and osteogenic ability of PPCs and CSCs is equal to that of BMMSCs, and PPCs are superior to MSCs for cartilage reconstruction *in vivo*. In addition, both PPCs and CSCs could produce sulfated glycosaminoglycan and collagenous components and maintain a non-calcified phenotype in the reconstructed cartilage. In Derks et al. (2013)’s study, porcine ear perichondrial progenitor cells (ePPCs), tracheal perichondrial progenitor cells (tPPCs), and BMMSCs were compared; the cells were induced in CMs for 4 weeks; and the results indicated that the expressions of collagen II, aggrecan, and cartilage oligomeric matrix protein in ePPCs are higher than those of tPPCs and BMMSCs. However, the expression of collagen I was comparable in all cell types, which showed that due to their higher chondrogenic potential and accessibility, ePPCs may be more convenient than tPPCs. Meanwhile, after comparing the differentiation ability of PPCs and CSCs, Xue et al. (2016) and Zhang et al. (2019) found that the cells differentiate into osteogenic lines, chondrogenic lines, and adipogenic lines under different induction conditions, and the preformation in these aspects of PPCs was better.

In Kagimoto et al. (2016)’s research, they verified that monkey PPCs could be induced into chondrocytes *in vitro* and regenerated into elastic cartilage by xenotransplantation into a nude mouse. For autologous transplantation, the monkey progenitor cells were developed into mature elastic cartilage in the subcutaneous region of a craniofacial section.

Since the formation of morphologically stable scaffold-free elastic cartilage tissue is challenging, Oba et al. (2022) developed a method for *in vitro* scaffold-free cartilage reconstruction. The use of human auricular PPCs significantly increased the potential for chondrogenesis by inducing chondrogenesis using microspheres similar to the ear colliculus. After craniofacial transplantation in nude mice, the size and elasticity of the reconstructed tissue remained unchanged, indicating that the reconstructed tissue was morphologically stable.

### Cartilage stem/progenitor cells

CSPCs are a type of stem cells found in human and animal ear cartilage, with the potential to differentiate into chondrocytes (Dowthwaite et al., 2004). The commonly used isolation methods for CSPCs include mechanical separation, enzymatic digestion, and magnetic bead sorting. Enzymatic digestion is currently the most commonly used method. By cutting ear cartilage tissue into small pieces, adding digestion enzymes (such as collagenase, pronase, or trypsin), and performing digestion on a constant temperature shaker at 37°C, single cells can be obtained. CSPCs have a differentiation ability that mainly tends toward chondrocytes. Studies have shown that, through appropriate inducers and culture conditions, CSPCs can differentiate into chondrocytes and synthesize cartilage matrix (Zucchelli et al., 2020). There is currently no unified standard for CSPC markers. However, researchers have discovered some markers associated with CSPCs, such as CD44 and CD90. The expression of these markers can help researchers identify and purify CSPCs (Kobayashi et al., 2011).

The main characteristics of the studies concentrating on CSPC differentiation analyzed are reported in Table 4.

**TABLE 4:** Main characteristics of the three studies included in the review on cartilage stem/progenitor cells.

Studies	Species of cells	Sorts of cells	Recipient animal	*In Vitro*/*in vitro*	Scaffold	Definition of auricular cartilage differentiation	Conclusion
Kobayashi et al. (2011)	Human	PPCs, CSPCs^a^	Mouse	*In vitro*/*in vitro*	—	Histopathological examination (HE^b^, Safranin O/fast green, alcian blue, toluidine blue stain, and Elastica van Gieson stain) and immunohistology (collagen types I and II)	This is a unique report demonstrating the presence of stem cells in auricular cartilage
Otto et al. (2022)	Human	PPCs, CSPCs	—	*In vitro*	—	Histopathological examination (Safranin O) and immunohistology (collagen type II)	Auricular cartilage progenitor cells demonstrate a potent ability to proliferate without losing their multipotent differentiation ability and produce a cartilage-like matrix in the 3D culture
Zucchelli et al. (2020)	Human	CSPCs	—	*In vitro*	—	Histopathological examination (HE, alcian blue, alcian blue/periodic acid—Schiff and Alizarin red)	In 3D spheroids, microtic and normal CSPCs undergo a chondrogenic differentiation process, which results in tissues morphologically similar to native microtic and normal cartilage, respectively. The similarity we have observed between microtic and normal CSPCs with their tissues of origin were not apparent in 2D cultures

^a^ CSPCs, cartilage stem/progenitor cells.

^b^ HE, hematoxylin and eosin.

In 2011, Kobayashi et al. first reported the presence of stem cells in auricular cartilage; they acquired the cells from human auricular perichondrium, and after the clonogenic progeny of a single CD44^+^ and CD90^+^, CSPCs demonstrated several features of stem cells (Kobayashi et al., 2011). Otto et al., 2022 isolated the cartilage stem/progenitor cells and cultured them in the 3D gelatin-based hydrogel *in vitro*, with subsequent biochemical, mechanical, and histological analyses. Auricular CSPCs showed strong proliferative capacity in 3D culture without losing their multipotent differentiation capacity and cartilage-like matrix production.

Zucchelli et al. (2020)’s study showed that in 3D spheroids, microtia and normal CSPCs underwent a chondrogenic differentiation process, which resulted in tissue morphology similar to that of native microscopic and normal cartilage, respectively. The discovery of CSPCs provided a new direction for stem cell differentiation; researchers can use the CSPCs from microtia cartilage to construct the cartilage scaffold for auricular reconstruction without any other extra resections.

In spite of the cells we previously demonstrated, another kind of cell was also used in auricular reparation. Eslaminejad and Bordbar (2012) found the blastema cells in rabbit ears and compared them with the BMMSCS. In adipogenic, osteogenic, and chondrogenic cultures, blastema cells expressed more lineage-specific genes than BMMSCs. They also multiply faster than BMMSCs *in vitro*.

### Stem cell differentiation method

Microtia is a significant challenge for plastic surgeons. In the past few decades, the use of the costal cartilage scaffold for auricular reconstruction has been dominant in such procedures. Nevertheless, the patients always have to suffer extra surgical incisions when harvesting the costal cartilage. The scaffold material for auricular reconstruction was very limited before application of the synthetic material. However, the synthetic material sometimes performed poorly (with low biocompatibility) in clinical applications; hence, the research to increase the biocompatibility of the scaffold material is becoming more and more vigorous. In recent years, the application of tissue engineering technology in reconstruction surgeries has increased dramatically. MSC/progenitor cells combined with or without those scaffold materials are being researched for producing durable ear cartilage replacements that conform to the functional and esthetic characteristics of the normal auricular function (Cao et al., 1997; Haisch et al., 2002; Shieh et al., 2004). To maintain the correct shape and elasticity of the tissue engineering scaffold after insertion under the skin, different strategies associated with culturing stem cells were invented.

Three main methods of stem cell differentiation were demonstrated in the studies we reviewed. The MSCs were injected directly into the cartilage niche to repair the defects of the auricular, where they were induced to differentiate into auricular cartilage in the physiological environment *in vivo*. Such kinds of cells can replicate and differentiate into different cell types, and the chondrogenic capacity of MSCs was improved (Raghunath et al., 2005; Robey and Bianco, 2006), and high-quality ear cartilage was formed *in vivo* (Bahrani et al., 2012). In this condition, chondrocyte growth was observed to go through a sequential phase from new, immature cartilage islands to mature, physically palpable cartilage plates, mimicking the formation of normal embryonic cartilage in many parts of the body, such as the auricle and nose. Although MSCs were observed to differentiate into auricular cartilage in the defective area of the ear in animal models, different kinds of MSCs also presented different capacities of chondrogenesis. In comparison, the researchers demonstrated that BMMSCs were superior to other stem cells in differentiation capacity (Chen et al., 2015; Oh et al., 2018; Hassan et al., 2022). BMMSCs are a representative cell source that promotes wound healing in multiple ways and develops into effector cells involved in angiogenesis, ECM formation, wound contraction, re-epithelialization, and matrix secretion (Hassan et al., 2022). This phenomenon was also observed in experiments in which stem cells were injected into cartilage defects. Compared to other induction methods, injecting MSCs directly into defect areas seems easier to implement, and since the regenerated cartilage only presents the properties of cartilage, it is not possible to further study the shape, quality, and support of the neo-cartilage.

The second main method of MSC induction was to co-culture the stem cells with the auricular cartilage cells *in vitro*. The results of co-culturing could be observed directly. In a further study, the mature cells were transplanted subcutaneously into animals for further *in vivo* observation. Since microtia cartilage is very limited in human beings and the development of microtia cartilage is congenital-insufficient, it is not enough to use microtia chondrocytes as the basis for ear cartilage culture. The microtia cartilage cells were isolated and co-cultured with MSCs. On one hand, those MSCs were induced into chondrocytes in a particular condition; on the other hand, a significant number of MSCs served as a complement to chondrocytes, increasing the total number of cells. In recent years, the co-culture of BMMSCs and chondrocytes has been developed to induce BMMSCs to form cartilage and inhibit cartilage hypertrophy (Yang et al., 2009; Fischer et al., 2010). The co-culture model also reduces the use of chondrocytes and makes it possible to obtain small pieces of cartilage for the repair of large defects. Comparison of the *in vitro* co-culture model with the BMMSC induction system alone showed that the co-culture of BMMSCs and chondrocytes reduced hypertrophy of tissue-engineered cartilage while enhancing its functional properties (Bian et al., 2011). After the co-culture model treatment, the cells were attached to the PLA/PCA scaffold, and the cartilage was formed *in vitro* and implanted into the subcutaneous tissue of the animal. After a period of observation, the elasticity and shape of the scaffold were well-maintained (Kang et al., 2012; Kang et al., 2013; Cheng et al., 2014; Zhang et al., 2014; Morrison et al., 2016; Zhao et al., 2017; Cohen et al., 2018; Dong et al., 2022). The results demonstrated that the co-culture model provided a high-quality strategy for auricular cartilage regeneration with significant potential for tissue-engineered auricular reconstruction.

Another method of stem cell induction was to culture the cells in a chondrogenic medium; the cells were induced in a particular medium *in vitro*, and some growth factors, antibiotics, and dexamethasone are added precisely to it (Cheng et al., 2014; Kagimoto et al., 2016). The MSCs could be induced into chondrocytes in these media; however, most of the chondrogenic ability of the cells was observed *in vitro*, although they showed good chondrogenic effects *in vitro* (Cheng et al., 2014; Kagimoto et al., 2016; Zhao et al., 2017; Otto et al., 2018). In a few *in vivo* experiments, Otto and Derks et al. found that cartilage induced *in vitro* could also show good morphology and type II collagen content after being implanted in animals for a period of time (Derks et al., 2013; Zhao et al., 2017); allogeneic stem cell-induced chondrocytes have also been studied as seed cells; the composite of functional chondrocytes and novel scaffolds can produce cartilage tissue after transplantation *in vivo*, due to the low immunity of cartilage; and the immune rejection of allogeneic functional cartilage transplantation is also weakened by the digestion, isolation, induction, *in vitro* culture, and carrier implantation of cartilage surface antigens. However, given that *in vivo* experiments are still in the preliminary stage of exploration, the *in vivo* transplantation effect of cartilage scaffolds prepared by this cartilage induction method needs to be further studied.

In addition to the aforementioned common induction methods, in recent years, stem cell exosomes, especially those derived from ADSCs, have been found to promote the differentiation of stem cells. Exosomes derived from tissue engineering can effectively promote the proliferation of microtia chondrocytes and the differentiation of mature cartilage (Chen et al., 2022). As a “cell-to-cell” messenger, ADSC exosomes have distinct characteristics and significant application potential in tissue regeneration by encapsulating various types of bioactive carriers. It can mechanistically play a role in different tissues by repairing specific functions such as cell migration and proliferation and promoting the formation of new blood vessels (Xiong et al., 2020). The research on stem cell-derived exosomes promoting ear cartilage regeneration and auricle reconstruction is a novel research area, which is more commonly used in wound healing, fat grafting, and articular cartilage reconstruction. The characteristics of stem cell exosomes that promote cell differentiation also provide direction for the future study of cell differentiation in ear reconstruction.

In the studies we reviewed, ADSCs, BMMSCs, PPCs, and CSPCs were the main stem cells that have been researched in craniofacial cartilage reconstruction, and each cell presented well-defined effects. The ADSCs were easily harvested and abundantly available in the body, given their well-known multipotent differentiation potential and the promoting effect of their exosomes on chondrogenic differentiation. ADSCs are a very important alternative stem cell in ear cartilage reconstruction. The BMMSCs also have multipotent differentiation ability and performed well in the chondrogenic assay of allografts. In the research comparing different MSCs’ chondrogenic abilities, BMMSCs were found to have the optimal chondrogenic capacity, as measured by cartilage morphology, elasticity, and AGA and collagen II content. PPCs and CPSCs are easily isolated from a donor site without ectopic tissue formation, such as calcifications or fibrous tissue formation, and have the advantage that they can be harvested *in situ* from the microtia without additional incisions. They also play an important role in chondrogenic differentiation, are more chondrogenic than ADSCs and BMMSCs, and have a good prospect in auricle reconstruction.

## Conclusion and prospects

In conclusion, auricle reconstruction is a difficult task, and recent advances in biological tissue engineering, and collaborations between stem cell biologists and clinicians, offer an opportunity for auricular cartilage constructs that resemble the human ear in shape, size, and flexibility. At present, stem cell reconstruction of the auricle is still in the initial stage of animal experiments, and transplantation experiments with such scaffolds in large animals are still lacking. Inducing MSCs to differentiate into chondrocytes to construct an auricular scaffold and implanting it subcutaneously in large animals for long-term *in vivo* experiments should be the future research direction. At the same time, scaffolds carrying chondrocytes should be further screened for future research. There is still a long way to go to realize stem cell reconstruction of cartilage scaffolds instead of autologous materials and apply it to clinical practice.
